# Dibromido(6,6′-dimethyl-2,2′-bipyridine-κ^2^ *N*,*N*′)zinc(II)

**DOI:** 10.1107/S1600536809039610

**Published:** 2009-10-07

**Authors:** Robabeh Alizadeh, Zeinab Khoshtarkib, Katayoon Chegeni, Amin Ebadi, Vahid Amani

**Affiliations:** a Damghan University of Basic Sciences, School of Chemistry, Damghan, Iran; b Islamic Azad University, Shahr-e-Rey Branch, Tehran, Iran; c Department of Chemistry, Jame Elmi Karbordi University, Aleshtar 1 Center, Aleshtar, Lorestan, Iran; d Department of Chemistry, Islamic Azad University, Kazerun Branch, Kazerun, Fars, Iran

## Abstract

In the title compound, [ZnBr_2_(C_12_H_12_N_2_)], the Zn^II^ atom is four-coordinated in a distorted tetra­hedral arrangement by an *N*,*N*′-bidentate 6,6′-dimethyl-2,2′-bipyridine ligand and two bromide ions. In the crystal, there are aromatic π–π contacts between the pyridine rings [centroid–centroid distances = 3.818 (3) and 3.728 (4) Å].

## Related literature

For related crystal structures containing a Zn atom coordin­ated by an *N*,*N*-bidentate bipyridine or phenanthroline-type ligand and two halide ions, see: Ahmadi *et al.* (2008 ▶, 2009 ▶); Alizadeh, Heidari, *et al.* (2009 ▶); Alizadeh, Kalateh, *et al.* (2009 ▶); Blake *et al.* (2007 ▶); Khalighi *et al.* (2008 ▶); Khan & Tuck (1984 ▶); Khavasi *et al.* (2008 ▶); Khoshtarkib *et al.* (2009 ▶); Lee *et al.* (2007 ▶); Marjani *et al.* (2009 ▶); Reimann *et al.* (1966 ▶); Wriedt *et al.* (2008 ▶).
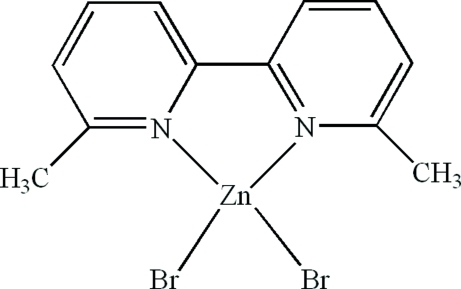


## Experimental

### 

#### Crystal data

[ZnBr_2_(C_12_H_12_N_2_)]
*M* *_r_* = 409.43
Monoclinic, 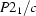

*a* = 7.6506 (15) Å
*b* = 10.279 (2) Å
*c* = 18.023 (4) Å
β = 95.93 (3)°
*V* = 1409.8 (5) Å^3^
*Z* = 4
Mo *K*α radiation
μ = 7.39 mm^−1^
*T* = 298 K
0.17 × 0.16 × 0.12 mm

#### Data collection

Bruker SMART CCD area-detector diffractometer
Absorption correction: multi-scan (*SADABS*; Bruker, 1998 ▶) *T*_min_ = 0.300, *T*_max_ = 0.418
11390 measured reflections
3822 independent reflections
2717 reflections with *I* > 2σ(*I*)
*R*_int_ = 0.068

#### Refinement

*R*[*F* ^2^ > 2σ(*F* ^2^)] = 0.058
*wR*(*F* ^2^) = 0.126
*S* = 1.11
3822 reflections
154 parameters
H-atom parameters constrained
Δρ_max_ = 0.81 e Å^−3^
Δρ_min_ = −0.68 e Å^−3^

### 

Data collection: *SMART* (Bruker, 1998 ▶); cell refinement: *SAINT* (Bruker, 1998 ▶); data reduction: *SAINT*; program(s) used to solve structure: *SHELXTL* (Sheldrick, 2008 ▶); program(s) used to refine structure: *SHELXTL*; molecular graphics: *ORTEP-3* (Farrugia, 1997 ▶); software used to prepare material for publication: *WinGX* (Farrugia, 1999 ▶).

## Supplementary Material

Crystal structure: contains datablocks I, global. DOI: 10.1107/S1600536809039610/hb5122sup1.cif

Structure factors: contains datablocks I. DOI: 10.1107/S1600536809039610/hb5122Isup2.hkl

Additional supplementary materials:  crystallographic information; 3D view; checkCIF report

## Figures and Tables

**Table:** 

Zn1—N1	2.071 (4)
Zn1—N2	2.072 (4)
Zn1—Br2	2.3400 (11)
Zn1—Br1	2.3444 (10)

**Table:** 

N1—Zn1—N2	80.22 (17)

**Fig. 1.**
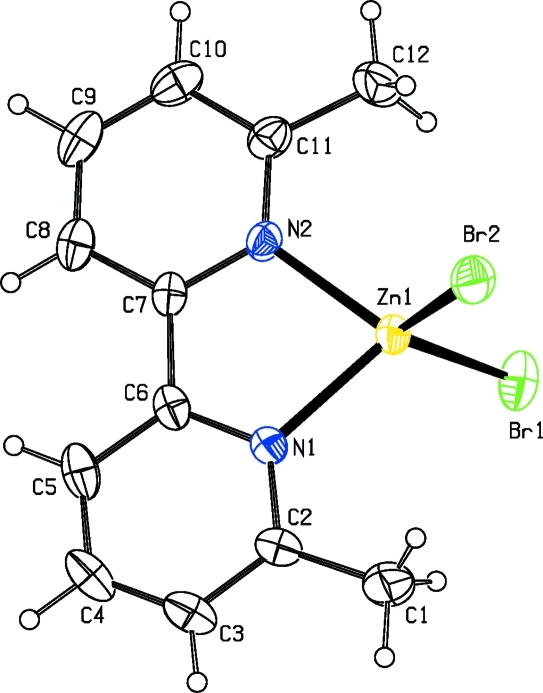
The molecular structure of (I) with displacement ellipsoids drawn at the 30% probability level.

**Fig. 2.**
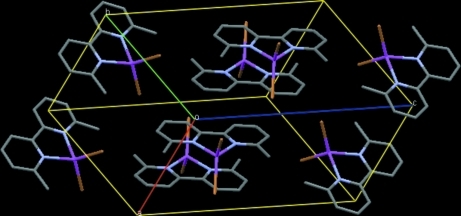
Unit-cell packing diagram for (I).

## supplementary crystallographic information

### Comment

Recently, we reported the synthes and crystal structure of [ZnCl_2_(phend)], (II), (Khoshtarkib *et al.*, 2009), [HgBr_2_(2,9-dmphen)], (III), (Alizadeh, Heidari *et al.*, 2009) and [Pb_4_(NO_3_)_8_(6-mbpy)_4_], (IV), (Ahmadi, Kalateh, Alizadeh *et al.*, 2009) [where phend is phenanthridine, 2,9-dmphen is 2,9-dimethyl-1,10-phenanthroline and 6-mbpy is 6-methyl-2,2'-bipyridine].

There are several Zn^II^ complexes, with formula, [Zn*X*_2_(N—N)], (*X* = Cl and Br), such as [ZnCl_2_(bipy)], (V), (Khan & Tuck, 1984), [ZnCl_2_(phen)], (VI), (Reimann *et al.*, 1966), [ZnCl_2_(dm4bt)], (VII), (Khavasi *et al.*, 2008), [ZnCl_2_(5,5'-dmbpy)], (VIII), (khalighi *et al.*, 2008), [ZnCl_2_(6-mbpy)], (IX), (Ahmadi, Kalateh, Ebadi *et al.*, 2008), [ZnCl_2_(6,6'-dmbpy)], (*X*), (Alizadeh, Kalateh *et al.*, 2009), [ZnCl_2_(PBD)]}, (XI), (Marjani *et al.*, 2009), [ZnBr_2_(4,4'-(dtbpy)].(Et_2_O), (XII), (Blake *et al.*, 2007), {ZnBr_2_[NH(py)_2_]},(XIII), (Lee *et al.*, 2007) and {ZnBr_2_[S(py)_2_]}, (XIV) (Wriedt *et al.*, 2008) [where bipy is 2,2'-bipyridine, phen is 1,10-phenanthroline, dm4bt is 2,2'-dimethyl-4,4'-bithiazole, 5,5'-dmbpy is 5,5'-dimethyl-2,2'-bipyridine, 6,6'-dmbpy is 6,6'-dimethyl-2,2'-bipyridine, PBD is *N*-(pyridin-2-ylmethylene)benzene-1,4-diamine, dtbpy is 4,4'-di-*tert*-butyl-2,2'-bipyridine, NH(py)_2_ is bis(2-pyridyl)amine and S(py)_2_ is bis(2-pyridyl)sulfide] have been synthesized and characterized by single-crystal X-ray diffraction methods. We report herein the synthesis and crystal structure of the title compound (I).

In the molecule of the title compound, (I), (Fig. 1), the Zn^II^ atom is four-coordinated in distorted tetrahedral configurations by two N atoms from one 6,6'-dimethyl-2,2'-bipyridine and two terminal Br atoms. The Zn—Br and Zn—N bond lengths and angles are collected in Table 1.

The π-π contacts between the pyridine rings, *Cg*1···*Cg*3^i^ and *Cg*2···*Cg*3^ii^ [symmetry cods: (i) 1-*X*,2-Y,-*Z*, (ii) –*X*,2-Y,-*Z*, where *Cg*1, *Cg*2 and *Cg*3 are centroids of the rings (Zn1/N1/C6—C7/N2), (N1/C2—C6) and (N2/C7—C11), respectively] further stabilize the structure, with centroid-centroid distance of 3.818 (3) and 3.728 (4) Å, respectively. It seems this π-π stacking is effective in the stabilization of the crystal structure (Fig. 2).

### Experimental

A solution of 6,6'-dimethyl-2,2'-bipyridine (0.20 g, 1.10 mmol) in methanol (10 ml) was added to a solution of ZnBr_2_ (0.25 g, 1.10 mmol) in acetonitrile (10 ml) and the resulting colourless solution was stirred for 20 min at at 313 K. This solution was left to evaporate slowly at room temperature. After one week, colorless prisms of (I) were isolated (yield 0.33 g, 73.7%).

### Refinement

All H atoms were positioned geometrically, with C—H = 0.93–0.96Å and refined as riding with *U*_iso_(H) = 1.2*U*_eq_(C) or 1.5U_eq_(methyl C).

### Crystal data

**Table:**

[ZnBr_2_(C_12_H_12_N_2_)]	*F*(000) = 792
*M**_r_* = 409.43	*D*_x_ = 1.929 Mg m^−^^3^
Monoclinic, *P*2_1_/*c*	Mo *K*α radiation, λ = 0.71073 Å
Hall symbol: -P 2ybc	Cell parameters from 1342 reflections
*a* = 7.6506 (15) Å	θ = 2.3–29.3°
*b* = 10.279 (2) Å	µ = 7.39 mm^−^^1^
*c* = 18.023 (4) Å	*T* = 298 K
β = 95.93 (3)°	Prism, colourless
*V* = 1409.8 (5) Å^3^	0.17 × 0.16 × 0.12 mm
*Z* = 4	

### Data collection

**Table:**

Bruker SMART CCD area-detector diffractometer	3822 independent reflections
Radiation source: fine-focus sealed tube	2717 reflections with *I* > 2σ(*I*)
graphite	*R*_int_ = 0.068
φ and ω scans	θ_max_ = 29.3°, θ_min_ = 2.3°
Absorption correction: multi-scan (*SADABS*; Bruker, 1998)	*h* = −9→10
*T*_min_ = 0.300, *T*_max_ = 0.418	*k* = −14→14
11390 measured reflections	*l* = −24→24

### Refinement

**Table:**

Refinement on *F*^2^	Primary atom site location: structure-invariant direct methods
Least-squares matrix: full	Secondary atom site location: difference Fourier map
*R*[*F*^2^ > 2σ(*F*^2^)] = 0.058	Hydrogen site location: inferred from neighbouring sites
*wR*(*F*^2^) = 0.126	H-atom parameters constrained
*S* = 1.11	*w* = 1/[σ^2^(*F*_o_^2^) + (0.0419*P*)^2^ + 2.306*P*] where *P* = (*F*_o_^2^ + 2*F*_c_^2^)/3
3822 reflections	(Δ/σ)_max_ < 0.001
154 parameters	Δρ_max_ = 0.81 e Å^−^^3^
0 restraints	Δρ_min_ = −0.68 e Å^−^^3^

### Special details

**Table:**

Geometry. All e.s.d.'s (except the e.s.d. in the dihedral angle between two l.s. planes) are estimated using the full covariance matrix. The cell e.s.d.'s are taken into account individually in the estimation of e.s.d.'s in distances, angles and torsion angles; correlations between e.s.d.'s in cell parameters are only used when they are defined by crystal symmetry. An approximate (isotropic) treatment of cell e.s.d.'s is used for estimating e.s.d.'s involving l.s. planes.
Refinement. Refinement of *F*^2^ against ALL reflections. The weighted *R*-factor *wR* and goodness of fit *S* are based on *F*^2^, conventional *R*-factors *R* are based on *F*, with *F* set to zero for negative *F*^2^. The threshold expression of *F*^2^ > σ(*F*^2^) is used only for calculating *R*-factors(gt) *etc*. and is not relevant to the choice of reflections for refinement. *R*-factors based on *F*^2^ are statistically about twice as large as those based on *F*, and *R*- factors based on ALL data will be even larger.

### Fractional atomic coordinates and isotropic or equivalent isotropic displacement parameters (Å^2^)

**Table:**

	*x*	*y*	*z*	*U*_iso_*/*U*_eq_	
C1	0.1523 (12)	0.9316 (8)	0.2403 (4)	0.077 (2)	
H1A	0.2661	0.8940	0.2538	0.092*	
H1B	0.0679	0.8635	0.2288	0.092*	
H1C	0.1185	0.9824	0.2812	0.092*	
C2	0.1591 (7)	1.0167 (6)	0.1736 (3)	0.0486 (13)	
C3	0.1234 (9)	1.1474 (7)	0.1764 (4)	0.0614 (16)	
H3	0.0946	1.1851	0.2204	0.074*	
C4	0.1306 (9)	1.2221 (6)	0.1134 (4)	0.0654 (18)	
H4	0.1055	1.3105	0.1144	0.078*	
C5	0.1755 (8)	1.1645 (6)	0.0485 (4)	0.0578 (15)	
H5	0.1801	1.2138	0.0054	0.069*	
C6	0.2132 (7)	1.0330 (5)	0.0485 (3)	0.0425 (11)	
C7	0.2666 (6)	0.9633 (5)	−0.0177 (3)	0.0388 (11)	
C8	0.2796 (8)	1.0242 (6)	−0.0855 (3)	0.0535 (14)	
H8	0.2510	1.1117	−0.0919	0.064*	
C9	0.3356 (9)	0.9530 (8)	−0.1428 (4)	0.0668 (19)	
H9	0.3455	0.9926	−0.1886	0.080*	
C10	0.3770 (8)	0.8238 (8)	−0.1333 (3)	0.0581 (16)	
H10	0.4156	0.7754	−0.1720	0.070*	
C11	0.3597 (8)	0.7663 (6)	−0.0638 (3)	0.0490 (13)	
C12	0.3987 (12)	0.6258 (7)	−0.0493 (4)	0.074 (2)	
H12A	0.2942	0.5824	−0.0371	0.089*	
H12B	0.4889	0.6177	−0.0084	0.089*	
H12C	0.4380	0.5868	−0.0930	0.089*	
N1	0.2039 (6)	0.9612 (4)	0.1102 (2)	0.0394 (9)	
N2	0.3051 (6)	0.8366 (4)	−0.0080 (2)	0.0390 (9)	
Zn1	0.26913 (8)	0.76759 (6)	0.09736 (3)	0.04134 (17)	
Br1	0.03105 (10)	0.62512 (7)	0.10150 (4)	0.0682 (2)	
Br2	0.52344 (8)	0.69964 (7)	0.17038 (3)	0.05791 (19)	

### Atomic displacement parameters (Å^2^)

**Table:**

	*U*^11^	*U*^22^	*U*^33^	*U*^12^	*U*^13^	*U*^23^
C1	0.119 (6)	0.070 (5)	0.045 (3)	0.017 (5)	0.023 (4)	−0.006 (3)
C2	0.048 (3)	0.047 (3)	0.051 (3)	0.006 (2)	0.007 (2)	−0.010 (2)
C3	0.064 (4)	0.052 (4)	0.067 (4)	0.014 (3)	0.002 (3)	−0.018 (3)
C4	0.071 (4)	0.036 (3)	0.085 (5)	0.012 (3)	−0.009 (4)	−0.013 (3)
C5	0.058 (3)	0.041 (3)	0.070 (4)	0.004 (3)	−0.014 (3)	0.015 (3)
C6	0.039 (3)	0.034 (3)	0.053 (3)	0.000 (2)	−0.003 (2)	0.004 (2)
C7	0.036 (2)	0.038 (3)	0.042 (3)	−0.004 (2)	−0.001 (2)	0.009 (2)
C8	0.057 (3)	0.054 (3)	0.048 (3)	−0.005 (3)	−0.001 (3)	0.017 (3)
C9	0.074 (4)	0.083 (5)	0.044 (3)	−0.013 (4)	0.008 (3)	0.020 (3)
C10	0.055 (3)	0.083 (5)	0.036 (3)	−0.003 (3)	0.005 (2)	−0.001 (3)
C11	0.055 (3)	0.056 (3)	0.036 (3)	−0.005 (3)	0.006 (2)	−0.002 (2)
C12	0.117 (6)	0.054 (4)	0.052 (4)	0.022 (4)	0.017 (4)	−0.006 (3)
N1	0.043 (2)	0.035 (2)	0.040 (2)	0.0048 (17)	0.0054 (18)	0.0015 (17)
N2	0.047 (2)	0.039 (2)	0.0310 (19)	−0.0009 (18)	0.0045 (17)	0.0043 (17)
Zn1	0.0537 (4)	0.0344 (3)	0.0365 (3)	0.0041 (3)	0.0078 (2)	0.0059 (2)
Br1	0.0718 (4)	0.0582 (4)	0.0742 (4)	−0.0162 (3)	0.0053 (3)	0.0207 (3)
Br2	0.0605 (4)	0.0634 (4)	0.0489 (3)	0.0125 (3)	0.0017 (3)	0.0138 (3)

### Geometric parameters (Å, °)

**Table:**

C1—C2	1.492 (9)	C8—C9	1.370 (10)
C1—H1A	0.9600	C8—H8	0.9300
C1—H1B	0.9600	C9—C10	1.372 (11)
C1—H1C	0.9600	C9—H9	0.9300
C2—N1	1.352 (7)	C10—C11	1.403 (8)
C2—C3	1.373 (8)	C10—H10	0.9300
C3—C4	1.376 (10)	C11—N2	1.340 (7)
C3—H3	0.9300	C11—C12	1.492 (9)
C4—C5	1.386 (10)	C12—H12A	0.9600
C4—H4	0.9300	C12—H12B	0.9600
C5—C6	1.383 (8)	C12—H12C	0.9600
C5—H5	0.9300	Zn1—N1	2.071 (4)
C6—N1	1.342 (7)	Zn1—N2	2.072 (4)
C6—C7	1.486 (8)	Zn1—Br2	2.3400 (11)
C7—N2	1.343 (7)	Zn1—Br1	2.3444 (10)
C7—C8	1.384 (7)		
			
C2—C1—H1A	109.5	C8—C9—C10	120.6 (6)
C2—C1—H1B	109.5	C8—C9—H9	119.7
H1A—C1—H1B	109.5	C10—C9—H9	119.7
C2—C1—H1C	109.5	C9—C10—C11	118.5 (6)
H1A—C1—H1C	109.5	C9—C10—H10	120.7
H1B—C1—H1C	109.5	C11—C10—H10	120.7
N1—C2—C3	120.8 (6)	N2—C11—C10	120.5 (6)
N1—C2—C1	117.8 (5)	N2—C11—C12	117.5 (5)
C3—C2—C1	121.4 (6)	C10—C11—C12	121.9 (6)
C2—C3—C4	119.4 (6)	C11—C12—H12A	109.5
C2—C3—H3	120.3	C11—C12—H12B	109.5
C4—C3—H3	120.3	H12A—C12—H12B	109.5
C3—C4—C5	119.5 (6)	C11—C12—H12C	109.5
C3—C4—H4	120.2	H12A—C12—H12C	109.5
C5—C4—H4	120.2	H12B—C12—H12C	109.5
C6—C5—C4	119.1 (6)	C6—N1—C2	120.5 (5)
C6—C5—H5	120.4	C6—N1—Zn1	113.7 (3)
C4—C5—H5	120.4	C2—N1—Zn1	125.8 (4)
N1—C6—C5	120.6 (5)	C11—N2—C7	120.4 (4)
N1—C6—C7	116.3 (4)	C11—N2—Zn1	125.8 (4)
C5—C6—C7	123.1 (5)	C7—N2—Zn1	113.8 (3)
N2—C7—C8	121.2 (5)	N1—Zn1—N2	80.22 (17)
N2—C7—C6	116.0 (4)	N1—Zn1—Br2	114.82 (13)
C8—C7—C6	122.8 (5)	N2—Zn1—Br2	115.86 (12)
C9—C8—C7	118.7 (6)	N1—Zn1—Br1	113.58 (12)
C9—C8—H8	120.7	N2—Zn1—Br1	114.84 (13)
C7—C8—H8	120.7	Br2—Zn1—Br1	113.53 (4)
			
N1—C2—C3—C4	1.1 (10)	C3—C2—N1—Zn1	178.0 (5)
C1—C2—C3—C4	−179.7 (7)	C1—C2—N1—Zn1	−1.2 (8)
C2—C3—C4—C5	−0.7 (10)	C10—C11—N2—C7	−0.2 (8)
C3—C4—C5—C6	−0.4 (10)	C12—C11—N2—C7	−179.5 (6)
C4—C5—C6—N1	1.0 (9)	C10—C11—N2—Zn1	179.2 (4)
C4—C5—C6—C7	−178.5 (5)	C12—C11—N2—Zn1	−0.1 (8)
N1—C6—C7—N2	−2.0 (7)	C8—C7—N2—C11	0.9 (8)
C5—C6—C7—N2	177.6 (5)	C6—C7—N2—C11	−178.0 (5)
N1—C6—C7—C8	179.2 (5)	C8—C7—N2—Zn1	−178.5 (4)
C5—C6—C7—C8	−1.3 (8)	C6—C7—N2—Zn1	2.6 (6)
N2—C7—C8—C9	−1.0 (9)	C6—N1—Zn1—N2	0.8 (4)
C6—C7—C8—C9	177.9 (6)	C2—N1—Zn1—N2	−177.8 (5)
C7—C8—C9—C10	0.3 (10)	C6—N1—Zn1—Br2	114.9 (3)
C8—C9—C10—C11	0.4 (10)	C2—N1—Zn1—Br2	−63.7 (5)
C9—C10—C11—N2	−0.5 (9)	C6—N1—Zn1—Br1	−112.2 (3)
C9—C10—C11—C12	178.8 (7)	C2—N1—Zn1—Br1	69.3 (5)
C5—C6—N1—C2	−0.6 (8)	C11—N2—Zn1—N1	178.7 (5)
C7—C6—N1—C2	179.0 (5)	C7—N2—Zn1—N1	−1.9 (4)
C5—C6—N1—Zn1	−179.3 (4)	C11—N2—Zn1—Br2	65.8 (5)
C7—C6—N1—Zn1	0.3 (6)	C7—N2—Zn1—Br2	−114.8 (3)
C3—C2—N1—C6	−0.4 (9)	C11—N2—Zn1—Br1	−69.7 (5)
C1—C2—N1—C6	−179.7 (6)	C7—N2—Zn1—Br1	109.7 (3)

## References

[bb1] Ahmadi, R., Kalateh, K., Alizadeh, R., Khoshtarkib, Z. & Amani, V. (2009). *Acta Cryst.* E**65**, m848–m849. 10.1107/S1600536809024180 PMC2977193 21583318

[bb2] Ahmadi, R., Kalateh, K., Ebadi, A., Amani, V. & Khavasi, H. R. (2008). *Acta Cryst.* E**64**, m1266. 10.1107/S1600536808028894 PMC2959229 21201019

[bb3] Alizadeh, R., Heidari, A., Ahmadi, R. & Amani, V. (2009). *Acta Cryst.* E**65**, m483–m484. 10.1107/S1600536809009994 PMC2977550 21583736

[bb4] Alizadeh, R., Kalateh, K., Ebadi, A., Ahmadi, R. & Amani, V. (2009). *Acta Cryst.* E**65**, m1250. 10.1107/S1600536809038215 PMC2970226 21577766

[bb5] Blake, A. J., Giunta, D., Shannon, J., Solinas, M., Walzer, F. & Woodward, S. (2007). *Collect. Czech. Chem. Commun.* **72**, 1107–1121.

[bb6] Bruker (1998). *SMART*, *SAINT* and *SADABS* Bruker AXS, Madison, Wisconsin, USA.

[bb7] Farrugia, L. J. (1997). *J. Appl. Cryst.* **30**, 565.

[bb8] Farrugia, L. J. (1999). *J. Appl. Cryst.* **32**, 837–838.

[bb9] Khalighi, A., Ahmadi, R., Amani, V. & Khavasi, H. R. (2008). *Acta Cryst.* E**64**, m1211–m1212. 10.1107/S1600536808027104 PMC2960607 21201646

[bb10] Khan, M. A. & Tuck, D. G. (1984). *Acta Cryst.* C**40**, 60–62.

[bb11] Khavasi, H. R., Abedi, A., Amani, V., Notash, B. & Safari, N. (2008). *Polyhedron*, **27**, 1848–1854.

[bb12] Khoshtarkib, Z., Ebadi, A., Alizadeh, R., Ahmadi, R. & Amani, V. (2009). *Acta Cryst.* E**65**, m739–m740. 10.1107/S160053680901959X PMC2969295 21582680

[bb13] Lee, Y. M., Hong, S. J., Kim, H. J., Lee, S. H., Kwak, H., Kim, C., Kim, S. J. & Kim, Y. (2007). *Inorg. Chem. Commun.* **10**, 287–291.

[bb14] Marjani, K., Asgarian, J., Mousavi, M. & Amani, V. (2009). *Z. Anorg. Allg. Chem.* **635**, 1633–1637.

[bb15] Reimann, C. W., Block, S. & Perloff, A. (1966). *Inorg. Chem.* **5**, 1185–1189.

[bb16] Sheldrick, G. M. (2008). *Acta Cryst.* A**64**, 112–122. 10.1107/S0108767307043930 18156677

[bb17] Wriedt, M., Jess, I. & Näther, C. (2008). *Acta Cryst.* E**64**, m315. 10.1107/S1600536808000081 PMC2960344 21201286

